# Adjunct rasagiline to treat Parkinson’s disease with motor fluctuations: a randomized, double-blind study in China

**DOI:** 10.1186/s40035-018-0119-7

**Published:** 2018-06-30

**Authors:** Zhenxin Zhang, Ming Shao, Shengdi Chen, Chunfeng Liu, Rong Peng, Yansheng Li, Jian Wang, Suiqiang Zhu, Qiumin Qu, Xiaoying Zhang, Haibo Chen, Xiangru Sun, Yanping Wang, Shenggang Sun, Baorong Zhang, Jimei Li, Xiaoping Pan, Gang Zhao

**Affiliations:** 1 Department of Neurology, Peking Union Medical College Hospital, 53 Dongdan N St, Dongcheng, Beijing, China; 2 Department of Neurology, The First Affiliated Hospital of Guangzhou Medical University, Guangzhou, China; 3 Department of Neurology, Ruijin Hospital, Shanghai Jiaotong University School of Medicine, Shanghai, China; 4 Department of Neurology, The Second Affiliated Hospital of Soochow University, Suzhou, China; 5 Department of Neurology, West China Hospital, Sichuan University, Chengdu, China; 6 Department of Neurology, Renji Hospital, Shanghai Jiaotong University School of Medicine, Shanghai, China; 7 Department of Neurology, Huashan Hospital, Fudan University, Shanghai, China; 8 Department of Neurology, Tongji Hospital of Tongji Medical College, Huazhong University of Science and Technology, Wuhan, China; 9 Department of Neurology, The First Affiliated Hospital of Xi’an Jiaotong University, Xi’an, China; 10 Department of Neurology, Beijing Tiantan Hospital, Capital Medical University, Beijing, China; 11 Department of Neurology, Beijing Hospital, Beijing, China; 12 Department of Neurology, Peking University First Hospital, Beijing, China; 13 Department of Neurology, The Second Affiliated Hospital of Guangzhou Medical University, Guangzhou, China; 14 Department of Neurology, Union Hospital, Tongji Medical College, Huazhong University of Science and Technology, Wuhan, China; 15 Department of Neurology, The Second Affiliated hospital of Zhejiang University School of Medicine, Zhejiang, Hangzhou China; 16 Department of Neurology, Beijing Friendship Hospital, Capital Medical University, Beijing, China; 17 Department of Neurology, Guangzhou First People’s Hospital, Guangzhou, China; 18 Department of Neurology, Xijing Hospital, The First Affiliated Hospital of The Fourth Military Medical University, Xi’an, China

**Keywords:** Parkinson’s disease, Rasagiline, Adjunct, Motor fluctuations, OFF time, Quality of life, China

## Abstract

**Background:**

The use of adjunct rasagiline in levodopa-treated patients with Parkinson’s disease and motor fluctuations is supported by findings from large-scale clinical studies. This study is to investigate the efficacy and safety of adjunct rasagiline in Chinese patients with Parkinson’s disease, as a product registration study.

**Methods:**

This 16-week, randomized, double-blind, parallel-group, multicenter, placebo-controlled study of rasagiline 1 mg/day included levodopa-treated patients with Parkinson’s disease and motor fluctuations. The primary efficacy endpoint was mean change from baseline in total daily OFF time over 16 weeks. Secondary endpoints were Clinical Global Impressions – Improvement (CGI-I), and change in Unified Parkinson’s Disease Rating Scale (UPDRS) Activities of daily living (ADL) and Motor scores. Patient well-being (EQ-5D), and the frequency of adverse events were also assessed.

**Results:**

In total, 324 levodopa-treated patients were randomized to rasagiline 1 mg/day (*n* = 165) or placebo (*n* = 159). Over 16 weeks, rasagiline statistically significantly reduced the mean [95% confidence interval] total daily OFF time versus placebo (− 0.5 h [− 0.92, − 0.07]; *p* = 0.023). There were also statistically significant improvements versus placebo in CGI-I (− 0.4 points [− 0.61, − 0.22]; *p* < 0.001), UPDRS-ADL OFF (− 1.0 points [− 1.75, − 0.27]; *p* = 0.008), and UPDRS-Motor ON (− 1.6 points [− 3.05, − 0.14]; *p* = 0.032) scores, as well as the EQ-5D utility index (*p* < 0.05). Rasagiline was safe and well tolerated.

**Conclusions:**

In levodopa-treated Chinese patients with Parkinson’s disease and motor fluctuations, adjunct rasagiline 1 mg/day statistically significantly reduced OFF time, and improved daily function and overall well-being, versus placebo. Consistent with findings in other countries, adjunct rasagiline was proven efficacious and well tolerated in Chinese patients.

**Trial registration number:**

NCT01479530. Registered 22 November 2011.

## Background

### Prevalence of Parkinson’s disease

Parkinson’s disease (PD) is a common, progressively disabling neurodegenerative disorder [1]. PD prevalence in China is estimated at 1.7% in individuals aged 65 or older– a rate comparable to that seen in European and other Asian countries [1, 2]. Moreover, the number of individuals over age 50 with PD in China is projected to rise to approximately 5 million by 2030 [3].

### Levodopa and motor fluctuations

For decades, levodopa has been the mainstay of therapy for PD and it is considered effective in relieving the symptoms of disease [4]. Nevertheless, a major problem associated with levodopa treatment is the appearance of motor complications including fluctuations in mobility [5, 6] and drug-induced dyskinesia [7–9].

Motor fluctuations have been reported in more than half of patients receiving levodopa after 5 years of treatment [10]. A multicenter survey in China found the overall prevalence rate for wearing-off in Chinese PD patients to be 46.5%, which is in line with reports from other countries [11]. Studies show that motor complications can appear relatively early, within a year of initiating levodopa [5, 12–14], and that the effectiveness of levodopa decreases with disease progression [6].

### Treatment with MAO-B inhibitors

Therapies beyond levodopa have been developed with the aim of avoiding, or at least controlling, motor fluctuations (including wearing-off) and dyskinesia, while also facilitating symptom control. Administration of a monoamine oxidase type B (MAO-B) inhibitor aims to prolong the availability and activity of dopamine in the brain – slowing the elimination of endogenous dopamine, as well as exogenous dopamine derived from levodopa [15]. In terms of clinical outcome, MAO-B inhibitor therapy has been associated with improvements in motor fluctuations [16, 17] and, in the long-term, a reduced risk of dyskinesia in patients with PD [18]. Moreover, the Chinese Parkinson’s Disease and Movement Disorder Society recommends MAO-B inhibitors for the management of wearing-off phenomena [19].

#### Rasagiline

Rasagiline is a potent MAO-B inhibitor [20]. Oral rasagiline has been approved for the treatment of idiopathic PD as monotherapy (without levodopa) or as adjunct therapy (with levodopa) in patients with end-of-dose fluctuations [21] in North America, Europe, and some Asian countries. The clinical efficacy, safety, and tolerability of rasagiline have been established in four Phase III studies (and one Phase II study) in predominantly Caucasian populations [16, 17, 22–24], and two Phase III studies in Chinese patients [25, 26]. Meta-analyses have reinforced these findings [27–29]. The safety and tolerability of rasagiline in the Chinese population has also been demonstrated in a preliminary study of a transdermal patch formulation [30].

### Rationale for the current study

To achieve product registration, the Chinese regulatory authorities require demonstration of efficacy and tolerability in a clinical trial conducted in the Chinese population [31]. The aim of the study was to evaluate the efficacy and safety of rasagiline (versus placebo) in levodopa-treated patients with PD and motor fluctuations, in a Chinese population.

Based on the submission, which included this study, rasagiline (Azilect®) received marketing authorization in China for the treatment of idiopathic PD as monotherapy (without levodopa) or as adjunct therapy (with levodopa) in patients with end of dose fluctuations (License No H20170336; 16 June 2017).

## Methods

The study was a randomized, double-blind, parallel-group, placebo-controlled, multicenter trial conducted in China *[Trial registration: NCT01479530].*

Patients were enrolled at 18 study sites (neurologist clinics) between 20th December 2011 and 3rd June 2013. Each study site was granted approval by the respective hospital’s ethics committee. Prior to enrolment, investigators provided patients with information about the study, and written, informed consent was obtained from all patients prior to any study-related activities.

The study was performed in compliance with the principles of Good Clinical Practice, and was designed and conducted in accordance with the principles of the Declaration of Helsinki [32].

### Patients

The study enrolled male and female, adult (≥30 years) Chinese outpatients with PD and motor fluctuations, who were receiving treatment with levodopa. Patients were selected from outpatient clinics, using competitive recruitment to encourage investigators to consider patients for the study (there was no fixed number per study site; rapid recruitment was encouraged; and study sites were remunerated on a per-patient basis).

Patient inclusion criteria were: diagnosis of idiopathic PD (at least two of resting tremor, bradykinesia, rigidity) without any other known or suspected cause of parkinsonism; optimized levodopa/dopa decarboxylase inhibitor (DDI) therapy, stable for ≥14 days prior to baseline (3–7 doses per day, not including bedtime dose); and ≥ 1 h/day of OFF time (during waking hours). Other main inclusion criteria were: modified Hoehn and Yahr score ≤ 3 in the ON state; stable dosing of any other anti-PD drug treatment for ≥30 days prior to baseline, and throughout the study; and an ability to keep accurate 24-h patient diaries. Main exclusion criteria were: cognitive impairment (defined as a Mini Mental State Examination, MMSE, score ≤ 24, i.e., at least mild cognitive impairment); melanoma or history of melanoma; previous neurosurgery for PD; presence of disabling dyskinesia; and known adverse reactions to tyramine-containing food. Disallowed concomitant medications included: MAO inhibitors (e.g., selegiline; within 90 days); antidepressants (within 42 days); sympathomimetics (including over-the-counter nasal or oral cold remedies); pethidine (within 14 days); and other psychotropic agents, including herbal (St John’s Wort, ephedra – ma huang).

### Study procedure

Informed consent was obtained during the screening visit. The screening period comprised 2 weeks of levodopa optimization (if required) and 2 weeks of stable levodopa treatment, followed by a 16-week double-blind treatment period, and a 4-week safety follow-up after study treatment.

During the screening period, patients were trained in the use of the ‘24-h’ diaries, after which they were required to be able to demonstrate ability to adequately complete three days of diaries before randomization took place. At baseline (start of the double-blind period), eligible patients were randomized (1:1) to receive either rasagiline 1 mg (one tablet, once daily), or matching placebo, taken preferably in the morning, for 16 weeks. The dose of concomitant levodopa could be adjusted (decreased by omitting one dose/extending dosing interval; or increased back up to baseline dosage) during the first 4 weeks (if required), but remained constant thereafter. Randomization was achieved using computer-generated (SAS®, Version 9.2) randomization numbers from the study sponsor (H. Lundbeck A/S), and a block (4) randomization method. Patients were randomized using an interactive voice/web response system (I*V*/*W*RS) hosted by Almac Clinical Technologies, Craigavon, United Kingdom. Study medication was supplied in wallet cards, with active and placebo treatment identical in appearance; the randomization code was not broken for any patient during the study. The study sponsor was responsible for study monitoring and other study-specific procedures.

#### Treatment compliance

At each visit after baseline, the patients were asked to return all the wallet cards, including unused investigational medicinal product (IMP). The investigator or his/her designated staff were responsible for accounting for all IMP dispensed to and returned by the patients. The investigator asked patients who withdrew from the study for the date of their last dose of IMP.

#### Assessments

The primary efficacy endpoint was change from baseline in mean total daily OFF time, as assessed by patient home diaries [33] averaged over visits at Weeks 4, 8, 12 and 16. Patients were trained and assessed in their use of the diary. The patient diary was divided into 30-min intervals, from which the patient selected one of four options for each interval: asleep; OFF; ON with no dyskinesia or without troublesome dyskinesia; or ON with troublesome dyskinesia. Following a 20-to 30-min training session, the patient was allowed to complete the diary if, in the investigator’s clinical opinion, the patient was able to assess their motor fluctuations correctly for a specified number of diary entries. During the screening visit, the patients completed 4 h (at least 8 diary ratings) of diary entries concurrently with, but independently from the investigator. There had to be at least 75% concordance in the diary entries between the patient and the investigator. The concordance had to include at least one ON to OFF, or OFF to ON, transition. The diary training session was to be repeated between the screening and baseline visits to achieve the 75% concordance requirement. For 3 days prior to each study visit, patients rated their status every 30 min as either ‘asleep’, ‘OFF’, ‘ON with no dyskinesia/without troublesome dyskinesia’ or ‘ON with troublesome dyskinesia’. OFF time values were averaged over the 3 days, and 90% of entries had to be filled in correctly for the diary to be considered acceptable (i.e., no more than 5 missing entries per day).

Secondary efficacy endpoints were the Clinical Global Impressions – Improvement (CGI-I) [34] scale score during ON time at Week 16, change from baseline to Week 16 in the Unified Parkinson’s Disease Rating Scale – Activities of daily living (UPDRS-ADL) score [35] during OFF time, and UPDRS-Motor score during ON time.

Overall health assessments included the EuroQoL 5-dimension (EQ-5D) [36], and the Parkinson’s Disease Questionnaire (PDQ-39) [37]. The scales used had been validated in the local language. The EQ-5D was assessed at baseline and at Weeks 2, 4, 8, 12 and 16; the PDQ-39 was assessed at baseline, Week 8 and Week 16.

#### Investigator and rater training

The study investigators were trained in the requirements of Good Clinical Practice at the investigators’ meetings or via an online system, and refresher training was provided. Rater training sessions were also conducted at the investigators’ meetings or on site. The study investigators were to be adequately experienced with patients with PD to rate patients using the UPDRS and Modified Hoehn and Yahr Staging. Only neurologists approved by the sponsor’s scales manager rated patients using the UPDRS and Modified Hoehn and Yahr Staging. MMSE was to be rated by a physician, and the CGI was rated by the investigator responsible for the patient. Only raters who qualified at a UPDRS rater qualification session were authorized to rate patients using the UPDRS.

Safety was assessed through the incidence of adverse events (AEs). Other safety endpoints included laboratory tests, vital signs, weight, electrocardiograms (ECGs), and physical and neurological examinations. Changes in UPDRS-Dyskinesia score (sum of items 32–34), and duration of ‘ON time with troublesome dyskinesia’ were also assessed in relation to safety.

### Statistical analysis

The sample size calculation was based on the primary endpoint using a two-sided significance test at the 5% level. Assuming a difference between rasagiline and placebo of 45 min in the mean change in total daily OFF time and a standard deviation (SD) of 2 h, a sample size of 150 patients per treatment group gave a power of 90%. Based on previous rasagiline studies, enrolment of 320 randomized patients was considered sufficient to secure 300 patients for the primary efficacy analysis.

Patient populations included the all-patients-randomized set (APRS), and all safety analyses were performed in the all-patients-treated set (APTS) of randomized patients who took at least one treatment dose. Efficacy was analyzed in the full-analysis set (FAS) of patients from the APTS who had at least one valid post-baseline measurement of total daily OFF time.

Primary and secondary endpoints were analyzed in a pre-planned hierarchical sequence: change in total daily OFF time (primary); CGI-I at Week 16; change from baseline in UPDRS-ADL OFF at Week 16; and change from baseline in UPDRS-Motor ON at Week 16. The testing continued from one endpoint to the next provided the difference from placebo was statistically significant in favor of rasagiline (*p* < 0.05) for that endpoint.

The primary efficacy endpoint was assessed using an analysis of covariance (ANCOVA) of the mean change from baseline in total daily OFF time (mean of changes over four study visits), with treatment and site as fixed factors and baseline total daily OFF time as a covariate, using observed cases (OC), as prespecified in the study protocol. Exploratory analyses included, change from baseline in mean total daily OFF time, analyzed for visits at Week 4, 8, 12, and 16 by an ANCOVA model using last observation carried forward (LOCF) and OC principles, with treatment and center as fixed factors and baseline mean total daily OFF time as a covariate. A sensitivity analysis was also performed for the change from baseline in total daily OFF time using mixed model for repeated measures (MMRM), OC. This included a fixed effect of visit and site, baseline total daily OFF time as a covariate, treatment-by-visit interaction, and baseline-by-visit interaction, using an unstructured covariance matrix. If diaries were missing or unacceptable at a post-baseline visit, the total daily OFF time for that visit was calculated based on the remaining completed or acceptable diaries. An acceptable diary was defined as one with no more than 5 erroneous entries between 6 am and midnight. The same criterion was applied in the calculation of total daily ON time. If there were missing values at some visits (whole diaries not done or deemed unacceptable), the post-baseline average was calculated based on the remaining completed or acceptable diaries.

Secondary endpoints were analyzed by ANCOVA using LOCF with treatment and site as fixed factors and the corresponding baseline assessment as a covariate, with the exception of the analysis of CGI-I, which used the Clinical Global Impressions – Severity (CGI-S) score at baseline. Missing items at each visit for the UPDRS-ADL score during OFF time were replaced by the mean of the non-missing items, provided that the number of non-missing items was ≥10; otherwise, the UPDRS-ADL score during OFF time for that visit was assigned a missing value. For the UPDRS-Motor score, the number of non-missing items had to be ≥20. Missing values were imputed using the last observed value immediately prior to the missing value. Data from the withdrawal visit were assigned to a nominal visit using visit windows. If two competing assessments had the same nominal visit, then the originally observed visit was kept for by-visit analyses, whereas the withdrawal visit was used for the LOCF analyses.

EQ-5D and PDQ-39 scores were analyzed by ANCOVA (OC), with treatment and site as fixed factors and the respective baseline score as a covariate. Missing items for the EQ-5D were not imputed. For the PDQ-39 dimensions, missing data were integrated in the scoring algorithm using the following rule: a dimension score was calculated only if ≥50% of its constitutive items had valid answers.

No interim analyses were planned.

Statistical analyses were conducted by H. Lundbeck A/S. The principal statistical software used was SAS®, Version 9.2.

## Results

### Baseline characteristics

**Fig. 1:**
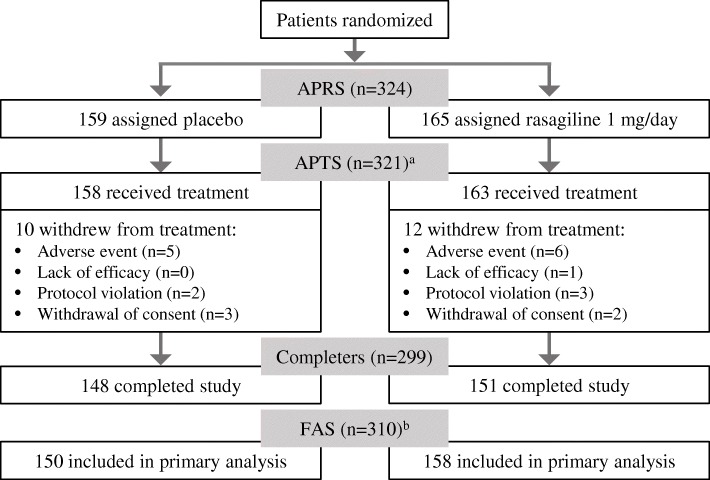
Flow of patients through the study.^a^3 patients did not receive treatment and were excluded from the APTS. ^b^11 patients did not have valid post-baseline diaries and were excluded from the FAS; 2 further patients (in the placebo group) were excluded from the primary efficacy analysis due to having no baseline diaries, but were in the FAS for all non-diary-related endpoints. APRS = all-patients-randomized set; APTS = all-patients-treated set; FAS = full-analysis set (modified intent-to-treat)

A total of 366 patients were screened, and 324 patients were randomized – as shown in Fig. 1 – and the overall withdrawal rate was low (22 patients; 7%). Patient demographics and clinical characteristics were similar between the two treatment groups at baseline (Table 1). The population was Asian (Chinese), with a mean age of 62 years (range 32–83 years), mean disease duration of approximately 7 years, and mean daily OFF time of 6.1 h.

**Table 1:** Patient demographics and characteristics at baseline (all-patients-treated set)

	Placebo (*n* = 158)	Rasagiline 1 mg/day (*n* = 163)
Age, years	61.7 (9.9)	62.7 (8.9)
Gender, male	109 (69%)	103 (63%)
BMI	23.2 (3.3)	23.1 (3.2)
Education (highest level)^a^		
Elementary/middle school	67 (44%)	68 (43%)
High school	45 (30%)	41 (26%)
College/university/graduate	33 (22%)	46 (29%)
None/other	7 (5%)	3 (2%)
Duration of PD, years	7.1 (4.3)	7.4 (4.8)
Modified Hoehn and Yahr ON	2.0 (0.7)	1.9 (0.7)
Patients with dyskinesia	31 (20%)	49 (30%)
UPDRS-Dyskinesia	0.35 (0.9)	0.49 (1.2)
ON time with troublesome dyskinesia, hours	0.55 (1.8)	0.67 (2.0)
Patients taking concomitant anti-PD medications:^b^		
Anticholinergics	20 (13%)	21 (13%)
Amantadine	58 (37%)	50 (31%)
Dopamine agonists^c^	102 (65%)	104 (64%)
COMT inhibitors^d^	31 (20%)	34 (21%)
*Data for FAS population*	(n = 152)	(n = 158)
Total daily OFF time, hours^e^	6.1 (2.7)	6.1 (2.6)
Total daily ON time, hours^e^	9.3 (2.5)	9.4 (2.4)
UPDRS-ADL OFF	16.5 (7.5)	15.6 (7.2)
UPDRS-ADL ON	7.3 (5.1)	6.8 (4.6)
UPDRS-Motor ON	25.6 (10.5)	23.8 (10.5)
CGI-S	4.1 (0.8)	3.9 (0.8)
Total daily levodopa dose, mg	550 (224)	501 (222)
Total daily levodopa dose at end of Week 4, mg^f^	549 (225)	495 (219)

^a^FAS population (*n* = 152 for placebo group, *n* = 158 for rasagiline group)

^b^medication that was continued after the first study treatment dose

^c^bromocriptine, piribedil, pramipexole, pramipexole dihydrochloride; ropinirole hydrochloride

^d^entacapone

^e^*n* = 150 for placebo group

^f^FAS, OC (*n* = 151 for placebo group, *n* = 157 for rasagiline group)

### Patient diaries and compliance with IMP

#### Patient diaries

A total of 174,816 post-baseline diary records were assessed, of which < 1% were considered unacceptable –placebo 0.4% (387/86,256); rasagiline 0.6% (532/88,560).

#### Compliance with IMP

The mean number of days of exposure to IMP was approximately 107 days in the placebo group and approximately 106 days in the rasagiline group. The majority of the patients received IMP for between 85 and 112 days (68% in the placebo and 76% in the rasagiline group). The mean compliance with IMP was ≥99% (placebo group: 86–100%; rasagiline group: 88–100%).

### Efficacy analyses

The FAS consisted of 310 patients, two of whom were excluded from all analyses of OFF and ON time (including primary analysis) as they had no diary entries at baseline.

#### Change in Total daily OFF time

**Fig. 2:**
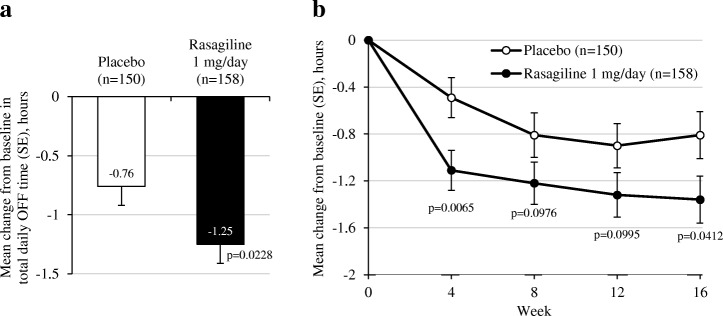
Changes from baseline in total daily OFF time. **a** Primary endpoint: mean change from baseline, averaged over visits at Weeks 4, 8, 12 and 16, in total daily OFF time (full-analysis set, analysis of covariance, observed cases). **b** Adjusted change from baseline in total daily OFF time (full-analysis set, analysis of covariance, last observation carried forward). SE = standard error

In the primary efficacy analysis, the mean change from baseline in total daily OFF time was statistically significantly greater in the rasagiline-treated group than in the placebo group, with a treatment difference of − 0.5 h, in favor of rasagiline (Fig. 2a, Table 2). Results of the exploratory analyses of mean change from baseline in total daily OFF time showed similar results to the primary efficacy analysis (Table 2). The ANCOVA OC and the MMRM sensitivity analysis were consistently in favor of rasagiline at all timepoints, although this reached statistical significance at Week 4 only in both analyses. Results of the ANCOVA, LOCF analysis were consistently in favor of rasagiline at all timepoints, and reached statistical significance at Weeks 4 and 16 (Fig. 2b**,** Table 2).

**Table 2:** Change from baseline in efficacy endpoints (full-analysis set)

	Adjusted mean change from baseline (SE)		Difference vs placebo (95% CI)	*p*-values
	Placebo (*n* = 152)	Rasagiline 1 mg/day (*n* = 158)		
Primary endpoint (mean change over Weeks 4, 8, 12, and 16; ANCOVA, OC)				
Total daily OFF time, hours^a^	− 0.76 (0.16)	−1.25 (0.16)	−0.50 (− 0.92, − 0.07)	0.023
Secondary endpoints (Week 16; ANCOVA, LOCF)				
CGI-I^b^	3.56 (0.07)	3.15 (0.07)	−0.41 (− 0.61, − 0.22)	< 0.001
UPDRS-ADL OFF	−1.20 (0.28)	− 2.21 (0.28)	− 1.01 (− 1.75, − 0.27)	0.008
UPDRS-Motor ON	−1.75 (0.55)	− 3.34 (0.55)	− 1.60 (− 3.05, − 0.14)	0.032
Exploratory analyses (mean change from baseline to Week 16 in total daily OFF time)				
ANCOVA, OC	−0.87 (0.21) (*n* = 147)	− 1.39 (0.21) (*n* = 151)	− 0.52 (− 1.06, 0.02)	0.0602
ANCOVA, LOCF	−0.81 (0.20)	− 1.36 (0.20)	−0.55 (− 1.08, − 0.02)	0.0412
MMRM, OC	−0.81 (0.20)	−1.34 (0.20)	− 0.53 (− 1.07, 0.00)	0.0514
PDQ-39 summary index and dimension scores (mean change, SE at Week 16; ANCOVA, OC)				
Summary index	−0.1 (0.8)	−1.9 (0.8)	−1.8 (−3.96, 0.42)	0.1122
Activities of daily living	2.0 (1.3)	−4.0 (1.3)	−6.1 (−9.52, − 2.64)	< 0.001
Bodily discomfort	1.7 (1.4)	− 2.2 (1.4)	−3.9 (− 7.65, − 0.12)	0.043
Cognition	0.9 (1.2)	0.4 (1.2)	−0.6 (−3.77, 2.67)	0.737
Communication	−1.0 (1.2)	0.1 (1.2)	1.1 (−2.09, 4.23)	0.506
Emotional well-being	−0.6 (1.5)	−3.0 (1.5)	−2.4 (−6.35, 1.49)	0.224
Mobility	−1.6 (1.2)	−4.1 (1.2)	−2.5 (−5.59, 0.53)	0.104
Social support	−1.2 (1.1)	0.4 (1.1)	1.6 (−1.35, 4.53)	0.288
Stigma	−2.2 (1.4)	−4.7 (1.4)	−2.5 (−6.22, 1.32)	0.202
EQ-5D (mean change, SE at Week 16; ANCOVA, OC)				
Utility index	0.00 (0.02)	0.05 (0.02)	0.05 (0.01, 0.09)	0.024
Health state^c^	0.77 (1.18)	5.09 (1.20)	4.31 (1.18, 7.45)	0.007

^a^*n* = 150 for placebo group

^b^absolute value at Week 16

^c^measured using visual analogue scale

#### Motor symptoms, daily function, and global outcomes

Tested in the defined hierarchical testing sequence, the primary and secondary endpoints were all statistically significantly in favor of rasagiline versus placebo (Table 2). Findings indicated that rasagiline produced statistically significant benefits versus placebo in overall global improvement (CGI-I), daily function during OFF time (UPDRS-ADL OFF), and motor symptoms during ON time (UPDRS-Motor ON) at Week 16.

#### Health-related quality of life

Rasagiline produced a statistically significant improvement versus placebo in the PDQ-39 dimensions of activities of daily living (− 6.1 ± 1.8 [standard error] points; *p* < 0.001) and bodily discomfort (− 3.9 ± 1.9 points; *p* = 0.043) (Table 2). The patients’ general well-being (EQ-5D utility index; 0.05 ± 0.02 points; *p* = 0.024) and perception of their own health state (EQ-5D visual analogue scale, VAS; 4.31 ± 1.59 points; *p* = 0.007) were also statistically significantly improved with rasagiline treatment versus placebo (Table 2).

### Safety analyses

The overall incidence of AEs was similar between the rasagiline (40.5%) and placebo (37.3%) groups (Table 3), with only one treatment-emergent adverse event (TEAE) occurring at a frequency of ≥5% in either group: dyskinesia was reported in 7.6% of placebo-treated patients and 6.7% of rasagiline-treated patients. The majority of TEAEs were mild or moderate, in both treatment groups. The rate of withdrawal due to AEs was low and similar between the rasagiline (3.7%) and placebo (3.2%) groups, as was the incidence of serious adverse events (SAEs) (4.3% for rasagiline and 3.2% for placebo) (Table 3). No deaths occurred during the study.

**Table 3:** Frequency of adverse events (all-patients-treated set)

	Number of patients (%)	
	Placebo (*n* = 158)	Rasagiline 1 mg/day (*n* = 163)
Patients with AEs	59 (37.3)	66 (40.5)
Patients with AEs leading to withdrawal	5 (3.2)^a^	6 (3.7)^b^
Patients with SAEs	5 (3.2)^c^	7 (4.3)^d^
Most frequent TEAEs^e^ (preferred terms; ≥1% in either group):		
Dyskinesia	12 (7.6)	11 (6.7)
Dizziness	0 (0.0)	8 (4.9)
Hypotension	0 (0.0)	6 (3.7)
Parkinson’s disease^f^	7 (4.4)	5 (3.1)
Alanine aminotransferase increased	1 (0.6)	3 (1.8)
Aspartate aminotransferase increased	1 (0.6)	3 (1.8)
Nausea	2 (1.3)	3 (1.8)
Vomiting	0 (0.0)	3 (1.8)
Fall	2 (1.3)	2 (1.2)
Hallucination	0 (0.0)	2 (1.2)
Headache	0 (0.0)	2 (1.2)
Herpes zoster	0 (0.0)	2 (1.2)
Insomnia	0 (0.0)	2 (1.2)
Pain in extremity	1 (0.6)	2 (1.2)
Rib fracture	0 (0.0)	2 (1.2)
Diarrhea	4 (2.5)	1 (0.6)
Abdominal distension	3 (1.9)	1 (0.6)
Nasopharyngitis	3 (1.9)	1 (0.6)
Accidental overdose	3 (1.9)	0 (0.0)
Constipation	3 (1.9)	0 (0.0)
Fatigue	2 (1.3)	1 (0.6)
Somnolence	2 (1.3)	1 (0.6)
Muscle spasms	2 (1.3)	0 (0.0)
Thrombocytopenia	2 (1.3)	0 (0.0)
URTI	2 (1.3)	0 (0.0)
Viral URTI	2 (1.3)	0 (0.0)

^a^Dyskinesia*, femur fracture, intervertebral disc protrusion, Parkinson’s disease*, psoriasis* (all *n* = 1)

^b^hallucination* (*n* = 2), dyskinesia*, epilepsy, hypotension*, transient ischemic attack (all *n* = 1)

^c^edema peripheral, erysipelas* + psoriasis*, femur fracture, intervertebral disc protrusion, multiple fractures + road traffic accident (all *n* = 1

^d^appendicitis, delusional perception*, Parkinson’s disease*, peripheral nerve injury, sick sinus syndrome, transient ischemic attack, venous stenosis (all *n* = 1)

^e^an event that started after the first dose of study treatment and prior to the last protocol-specified contact with that patient

^f^patients reported symptom aggravation/disease progression

^*^Considered by the investigator to be possibly or probably related to treatment

There were no clinically relevant changes in clinical safety laboratory values, vital signs, weight, or ECG status. The mean (SD) change in UPDRS-Dyskinesia score was slight: placebo, 0.05 (0.59) points; rasagiline, 0.13 (0.94) points. The mean duration of ON time with troublesome dyskinesia remained relatively unchanged from baseline to Week 16 in the placebo (0.55 and 0.65 h, respectively) and rasagiline groups (0.67 and 0.87 h, respectively).

## Discussion

Building on the findings of two large-scale registration studies (LARGO and PRESTO) set in Europe/America [16, 17], the current study investigated the efficacy and safety of rasagiline 1 mg/day as adjunct to levodopa in a Chinese population of patients with PD and motor fluctuations. Although rasagiline had been extensively studied, with proven efficacy and tolerability [38], for product registration purposes the Chinese regulatory authorities require a further study in a Chinese population to support marketing authorization in China. Thus, the study reported here was undertaken to support the marketing authorization submission for rasagiline as adjunct treatment (with levodopa) for patients with idiopathic PD and end-of-dose fluctuations – and adds to the overall clinical evidence base for rasagiline.

The primary efficacy analysis of reduction in mean total daily OFF time revealed a statistically significant difference of 30 min between the rasagiline and placebo groups. The effect of rasagiline in reducing daily OFF time (− 1.25 h) was comparable to that seen in Caucasian patients in the LARGO study (− 1.18 h), although the placebo response in the current study was greater than in LARGO (− 0.76 h compared with − 0.40 h) [16]. The placebo response seen here was also greater than that seen in a similar study of rasagiline in Chinese patients (− 0.69 h) although, in that study, there was a greater reduction in OFF time (− 1.75 h) associated with rasagiline treatment [25]. The occurrence of a placebo response in randomized clinical studies is well documented, prominent in PD [39], and has been observed in both Caucasian and Asian populations. Placebo response in PD has been linked with an increased endogenous dopamine release (and resulting symptom alleviation), possibly related to a good doctor–patient relationship, and the patient’s expectation of clinical benefit (involving reward learning enhancement and the appraisal network) [39–42]. A South Korean study of adjunct entacapone in patients with wearing-off phenomena, reported a placebo response of approximately a 1-h reduction in daily OFF time [43] – a magnitude of effect comparable to that observed in our study.

In a 12-week study of PD patients with motor fluctuations, conducted in various geographical regions at approximately the same time as our study in Chinese PD patients, rasagiline 1 mg/day (included as active comparator), despite reducing daily OFF time by more than 1 h, failed to demonstrate statistically significant separation from placebo in total daily OFF time (rasagiline − 1.1 h, placebo − 0.8 h, *p* = 0.28). [44] The observed large placebo response was considered to be a factor contributing to the failure of the study, as were various study design and conduct issues. [44] Regardless of patient ethnicity, the results of our study presented here serve to reconfirm the efficacy of rasagiline as adjunctive therapy in patients with PD experiencing motor fluctuations.

Concurrent with the reduction in OFF time, statistically significant improvements in the secondary efficacy endpoints (CGI-I score, UPDRS-ADL score during OFF time, and UPDRS-Motor score during ON time) were also observed with rasagiline, which supported the results of the primary efficacy analysis.

Results of the primary and secondary analyses also correlated with meaningful improvements in overall health and the patient’s perception of their own well-being. The improvements in PDQ-39 scores in the rasagiline group indicated that these patients had a slightly better quality of life than those in the placebo group, with statistically significant benefits in the dimensions of ADLs and bodily discomfort.

It is notable that there was a lower UPDRS-Dyskinesia score (0.35–0.49, i.e., less severe symptoms) at baseline in this Chinese study group, compared to that recorded in the LARGO study (1.4–1.5) [16]. This could result from the much lower daily dosage of levodopa (501–550 mg) in the Chinese study population compared to that of the LARGO study (697–722 mg), since the severity of disease, and concomitant use of anti-PD medications, at baseline are comparable between the two study populations [16]. Lower dose of levodopa at baseline (515–521 mg) was also seen in the Zhang et al. (2013) study of rasagiline in Chinese patients [25]. Additionally, a large cross-sectional survey in four urban regions of China revealed that many Chinese PD patients are treated with low-dose levodopa – medical practice that might be influenced by Chinese culture [45].

It was also observed that the improvements in motor symptoms/control (related to an improved supply of dopamine) were not accompanied by a worsening of dyskinesia in this Chinese study. At baseline, more patients in the rasagiline group suffered from dyskinesia compared with the placebo group. However, during the study the reporting of dyskinesia as a TEAE was similar between the placebo (7.6%) and rasagiline (6.7%) groups, and there were only small changes in UPDRS-Dyskinesia score, and in the duration of ‘ON time with troublesome dyskinesia’ (patient diaries), from baseline to Week 16. These results were consistent with a cohort study, which showed that MAO-B inhibitor therapy was associated with reduced risk of dyskinesia in patients with PD [18].

Overall, treatment with rasagiline was safe and well tolerated, with AEs reported at similar levels to the placebo group, and no new safety concerns observed. Indeed, safety findings were comparable to those of the (predominantly European) LARGO study [16] in terms of the incidence of dyskinesia, and the low incidence of withdrawals due to AEs. The findings are also consistent with existing study data demonstrating that the pharmacokinetics (and associated tolerability) of rasagiline were similar in Chinese and Caucasian populations [46].

Given that patients with PD require long-term therapy, a potential limitation of this study is the short duration of treatment (4 months). The effect of rasagiline in Chinese PD patients could be examined further in a long-term trial.

## Conclusions

In conclusion, rasagiline statistically significantly reduced OFF time in Chinese patients with PD and motor fluctuations. Despite the impact of a larger than expected placebo response, the 30-min reduction in disabling OFF time per day in the rasagiline group is notable. In addition, improvements were observed in the secondary and quality of life/general well-being endpoints in the rasagiline group, which support the primary endpoint. Consistent with data from similarly robust, well-controlled studies in other world locations, data from this Chinese study show that rasagiline is well tolerated, and a clinically useful adjunct therapy to levodopa in patients with PD.

## Abbreviations

ADL: Activity of daily living
AE: Adverse event
ANCOVA: Analysis of covariance
APRS: All-patients-randomized set
APTS: All-patients-treated set
BMI: Body mass index
CGI-I: Clinical Global Impressions – Improvement
CGI-S: Clinical Global Impressions – Severity
CI: Confidence interval
COMT: Catechol-*O*-methyltransferase
DDI: Dopa decarboxylase inhibitor
ECG: Electrocardiogram
EQ-5D: EuroQoL 5-dimension
FAS: Full-analysis set
IMP: Investigational medicinal product
I*V*/*W*RS: Interactive voice/web response system
LOCF: Last observation carried forward
MAO-B: Monoamine oxidase type B
MMRM: Mixed model for repeated measures
MMSE: Mini Mental State Examination
OC: Observed cases
PD: Parkinson’s disease
PDQ-39: 39-item Parkinson’s Disease Questionnaire
SAE: Serious adverse event
SD: Standard deviation
SE: Standard error
TEAE: Treatment-emergent adverse event
UPDRS: Unified Parkinson’s Disease Rating Scale
URTI: Upper respiratory tract infection
